# Animal Effluvia and Their Effects on Health

**Published:** 1855-12

**Authors:** R. D. Grainger


### TRANSACTIONS

OF THE

### EPIDEMIOLOGICAL SOCIETY OF LONDON.

### Papers and Communications.

### ANIMAL EFFLUVIA AND THEIR EFFECTS ON HEALTH.

By R. D. Grainger, Esq., F.R.S.

[*Read before the Epidemiological Society, on November* 5, 1855, *as the Introductory Address on the opening of the Session.*]

Mr. President and Gentlemen,—

In accordance with the request of your Council, I have the honour of addressing you on the occasion of thisthe opening of the sixth session of the Epidemiological Society. I undertake this office deeply impressed with the importance attaching to all the proceedings of our Association, relating as they do to a class of diseases, the zymotic, which are at once the most destructive and the most preventible ; which in this metropolis destroy at least one-fourth of all who die, and under the most unfavorable conditions, as, for example, in the district called the Potteries, Kensington, even one-half ; and which yet, by enlightened sanitary measures, may, as we have demonstrative evidence in the great experiment of the model establishments of this city, be almost extirpated.

If there be one peculiarity more than another by which physiological science is in the present day distinguished, it is by the discovery and recognition of the enormous influence exerted in the animal economy by the varied physical agents, by which we are surrounded. So long as the vague and misleading notions of the vital principle, as set forth by so many distinguished authorities, prevailed; when all the actions of the animal body, even including those which it is now certainly known depend on chemical or mechanical forces, were referred to the mysterious operations of life, it was natural to regard pestilential diseases, especially when presented in all their fearful intensity, as visitations equally beyond human scrutiny and human control. Even when more intelligent ideas began to prevail, more importance was attached to certain unknown but assumed meteorological phenomena, than to those more obvious and recognisable conditions, which, by the extended and accurate researches of late years, have been demonstrated to be the great developers of epidemic disease. I need not point out to you, sir,—to whom by a wisely directed choice English readers are indebted for the historical record of the great pestilences of former ages,—that epidemic disease is the special scourge of ignorant and barbarous nations, and that in the ratio of their darkness. Nor is it necessary to dwell upon what is nevertheless a deeply instructive truth, that in regard to physical existence, the one great distinction between civilized and savage, or even partially enlightened nations, as those of the east, is the total neglect among the latter of all the decencies of life comprised in the one word cleanliness. What the habitual existence of these people is, and what are the fearful penalties consequently inflicted on them, have been graphically described by English and French physicians acquainted with the east, of which the account of the Pali pestilence in India, and of the plague in Alexandria, may serve as types. That this violation of the fixed conditions of human existence, so universal among heathen and misbelieving nations, is one of the many penalties of our fallen nature, may, among other reasons, be inferred from this, that to guard the chosen people of God from like evils, a direct revelation of the Divine will in this respect was vouchsafed. To the sanitary reformer nothing can be more striking, nay even startling, than the precise and comprehensive laws laid down in the Mosaic books. No unclean thing was to remain within the dwellings of the theocratic nation; the refuse of slain animals was to be burnt without the camp; and the defilement of human excreta was forbidden under awful penalty, even the withdrawal of the divine presence. It is only now, after the lapse of nearly four thousand years since this the great sanitary code was proclaimed, that we are beginning to apply it in the polluted courts and alleys of our great cities.

These considerations may not perhaps be deemed foreign to the present occasion, inasmuch as the effluvia proceeding from putrid animal matter which I propose to notice, are precisely those abounding in the hovels of the Hindoo and in the mosque of the Mahometan, which last we are assured, in a report to the Academy of Medicine, a stranger can in lower Egypt discover by the fœtid air as he approaches.

In proceeding to the more immediate subject I am anxious to bring before the notice of the Society this evening, I may state that three considerations have more particularly determined my choice.

In the first place, it has always appeared to me, that as in populous places the alleged causes of preventible sickness are commingled together, it is essential they should be examined one by one, so as to obviate all incertitude with respect to each. In a former communication to this Society, I endeavoured to illustrate separately the noxious influence of two of the most universal sanitary evils,—overcrowding and a privy atmosphere; this evening I propose to do the same in regard to other forms of animal effluvia, and to illustrate their action on the economy; a second inducement has been the wide spread existence of such emanations, wherever human beings are aggregated together, whether in cities or in camps. And, in the last place, I am not unmindful that with respect to this particular class of agents, there is probably a greater conflict of opinion than in what concerns any other branch of sanitary science.

In order to obtain a right conception of the present question, some remarks on the general influence of putrid matter on the system would appear desirable. That, when in a concentrated form, such matter directly finds an entrance into the living body, as by a puncture in dissection, the most serious or even fatal results are the consequence, needs not be insisted on in this audience. There is, however, one consideration which presents itself in these often distressing cases; it is the inconceivable minuteness of the noxious agent, which is competent to produce the most destructive consequences: a puncture with the point of a needle, so fine that a lens is required to detect its existence, has sufficed in a few hours to destroy life. And such facts as these should be kept in remembrance, since nothing is more common among the general public, and occasionally in our own profession, than to hear doubts expressed whether the alleged predisposing causes of epidemics, such as emanations from surcharged burial grounds, or the effluvia from a knacker's yard, or other unhealthy trades, can have any real influence, because they are diluted in the atmosphere, and consequently are small in amount. It would be absurd to deny that there are great powers of resistance in the animal economy; but seeing how vigilantly nature provides in her eternal laws for the prevention of any, the least fractional deterioration of the atmosphere, in what, in contradistinction to the perverse interposition of man, may be called its normal constitution; how, for example, by an interposing intelligence, which even compels a general law to yield to a designed end, the heavy and poisonous carbonic acid, generated on the earth's surface by respiration and combustion, is compelled to ascend into the lighter atmospheric ocean; seeing these and a thousand similar instances, who can doubt that it is of supreme import to the maintenance of normal health, that the air we respire in such enormous volumes, thirty-six hogsheads daily, should be void of all, the least contamination. It may indeed be difficult clearly to demonstrate this under ordinary circumstances; but let a great epidemic come, and then scrutinize what takes place. It would be tedious to detail the accumulated proofs how, during cholera, when, as one has repeatedly seen, a feather will turn the balance, and even in less virulent epidemics, as gastric fever, severe localised and individual attacks have been directly traced to one or other of those so-called trifling atmospheric deteriorations.

Another and a most frequent mode of the introduction of decomposing animal matter, is in a more diffused form by way of the alimentary canal. The poisonous effects of putrid food, especially veal, pork, bacon, and cheese, are too familiar to require illustration; and late researches have shown how extensively the inhabitants of populous districts suffer from the use of water polluted by animal matter. There is, as it would appear, ample evidence of the truth of the opinion held by the great majority of observers, that, however introduced, all these substances find their way into the blood, a position of which it is impossible to exaggerate the importance, as lying at the bottom of the great doctrine of blood diseases, as applicable to epidemics, and which in latter years has received so vast, and, as it would appear, so legitimate as extension.

In attempting to trace the actual effects of putrid animal matter in a gaseous form, we encounter a difficulty on the very threshold of the inquiry; for, whereas, one of the first things one wishes to know is, of what do these effluvia, of which we hear so much, consist? it must be confessed that at present our knowledge on this prime point is most restricted; that is, so far as the ultimate nature of these agents, as determined by chemical analysis, is concerned. Our ignorance on this point is not, however, such as to exclude some very important considerations. In the first place, these effluvia must be well distinguished from the gases, often fœtid—the products of decomposition—by which they are, for the most part, accompanied. These gaseous matters are themselves often poisonous, as the most universal one, sulphuretted hydrogen; or, if sufficiently concentrated, irrespirable, as carbonic acid; they may even rapidly destroy life, but, if they do so, it is in a certain recognized manner, for the most part peculiar to each, and distinct from death connected with the class of agents we are considering. The earlier with the later inquirers, Fourcroy and Berzelius, equally with Riecke and Tardieu, have recognized the distinction and its importance.

Another certain inference is that effluvia consist of organic matter, a protein compound, in a most minutely divided form; therefore adapted, as we know, from the action of gaseous poisons, in an especial manner to enter the blood, and there to diffuse itself rapidly and extensively. As these subtle agents are then material and organic; as they are produced under certain known conditions and from certain sources, there is no reason why, as our means of investigation become more perfect, the exact nature, operation, and laws of effluvia should not be as completely known as those of any other class of substances.

Having submitted these general remarks, I would more specifically notice some of the more striking instances of putrid emanations with the view of inquiring how far they operate injuriously on the living body. When in a concentrated form, and marking their presence by a fœtid odour, the common judgment of mankind, in these days and among civilized nations, pronounces all putrescent effluvia to be noxious, and, under unfavourable circumstances, highly pestilential. It is indeed somewhat strange, that the arguments it is the lot of the sanitary reformer to encounter, when the question of removing some offensive trade comes to be discussed, often are made to rest on the opinion of a few members of our own profession in this and other countries, who contend, that the putrid atmosphere arising from the decomposition either of human bodies, as in the dissecting room or field of battle; or of the carcases of animals, as in the celebrated, but now abolished depôt of Montfaucon, are essentially innocuous.

It would be impracticable, on an occasion like this, to enter on so wide a question; and, therefore, I only propose briefly to notice two or three of the best marked examples of these effluvia, remarking, *en passant*, that the question as to thousands of unburied, or partially buried, bodies of men and animals, in immediate proximity to a great camp and tainting the air all around, has practically been long since settled by common consent; and that the only thing now engaging the most anxious solicitude of the governments, unhappily engaged in war, is to know what is the most efficient mode of preventing the escape of these fœtid, poisonous emanations, to insure which no outlay is considered too costly, no efforts too great.

As coming more immediately under the cognizance of all present, I would, in the first place, inquire what is the influence of the dissecting-room; is it, or is it not, noxious as regards those who frequent it? For myself I may say that, in the course of a somewhat extended experience, I never heard from those actually engaged in practical anatomy but one opinion. But as the views to which I have above alluded are from time to time reproduced, it appeared to me worth while to obtain the experience of some friends who have been demonstrators of Anatomy at King's College, Guy's, the Middlesex Hospital and elsewhere. The general results of my inquiries are these: that of the students who work in the dissecting-room many suffer from diarrhœa at the outset; that some young men never become habituated, always suffering from diarrhœa on resuming dissection; in some limited cases vomiting is induced, and in some hard workers nervous depression, pallor, boils, and relaxed and ulcerated throat have been produced. Since this was written my friend Professor Owen, whose opinion must have great weight, inasmuch as in addition to his habits of profound observation, he has in an official capacity had occasion to investigate the influence of animal effluvia, has favoured me with his opinion on the point in question. He writes as follows:—"My experience of dissecting-room influences, when an anatomical student, strongly impressed me with the poisonous effects of its atmosphere: few escaped without some seasoning attack of diarrhœa; some students of less careful habits succumbed yearly. I attribute my own endurance of a good proportion of time spent in that atmosphere, to my careful and abstemious diet."

So far as I have inquired, fever, as a direct consequence, has not been traced; though I have often known students attacked by it, who had spent much time in the dissecting room. My friend Mr. Whitfield, resident medical officer of St. Thomas's Hospital, informs me he has repeatedly attended students, labouring under severe diarrhœa attended with febrile disturbance, the result of dissection. It must be also recollected, that great improvements as to ventilation, and the free use of disinfectants, have of late years been introduced; and that dissection in the hot summer weather has been abandoned. Knowing by extended experience how actively diarrhœa predisposes to cholera, no one free from preconceived ideas, would doubt that the prosecution of anatomy in an epidemic season would endanger health. It may, indeed, seem almost superfluous to insist upon a point all but universally recognised.

As an assumed proof of the innocuousness of putrid effluvia, the healthy condition of butchers has often been adduced, and by distinguished members of our own profession. Those, however, who have actually investigated the matter, have arrived at very different conclusions; for, whilst it is true that the ordinary journeymen whom we see in the shops are a healthy class, being little occupied in the slaughter-house, and much in the open air, those engaged by what are called carcase butchers, and whose whole occupation is slaughtering, are found to be unhealthy. Professor Owen, who had occasion to report on this subject, states that these slaughtermen had sallow, tallowy complexions, and a general unhealthy aspect, the causes being the habitual respiration of a foul atmosphere, and the addiction to the use of stimulants, resorted to as a remedy for the depressed nervous power induced, as it appeared, by the unfavourable, but improveable, circumstances under which they had to gain their daily bread.

I now, sir, come to speak of a class of effluvia to which much attention has of late years been directed, and which, as connected with important legislative enactments, are of special interest: I refer to the emanations proceeding from burial grounds. We are here again met with conflicting opinions. There is no doubt a difficulty, as in other causes of unhealthiness, in tracing the direct and specific influence of the assumed agent.

The celebrated treatise of Dr. Riecke, *On the Influence of Putrid Air and Burial Grounds*, and the more recent work of M. Tardieu, entitled *Voiries et Cimetières*, abound in such remarkable instances, in which either death or serious illness was directly caused by exposure to the effluvia proceeding from corpses recently interred, as to leave no room for doubt as to the origin of the mischief. One such instance, recorded by Vicq d'Azyr, may serve as an example. "A person of distinction having died in December 1773, a large vault was prepared in a church; and, in making this, it was necessary to remove several corpses, and among the rest, one that had been interred in the preceding February. Putrid effluvia spread in the church; but this did not prevent the funeral. Of those who assisted at the obsequies, fifteen perished within eight days; among whom were four peasants, who had prepared the vault and removed the coffins."

The following case, related by Maret, is perhaps more interesting, and closely corresponds with an instance that fell within my own knowledge: "A very corpulent man was buried in a parish church near Dijon. Owing to mismanagement, the coffin was only covered with a foot of earth, and a tomb-stone seven inches thick. In a few days, cadaverous emanations infected the air, till, at the end of three weeks, the church was obliged to be abandoned. It was then determined to remove the corpse, and three grave-diggers were employed; two of them could not resist the fœtid vapours, were attacked with nausea and violent vomiting, and refused to continue the work. The third man persevered, and achieved the removal of the body; he became, however, so ill that he had difficulty in getting home; he vomited repeatedly, took fever, and died at the end of ten days."

In the course of the official inquiries I have had to make in respect to intramural burial, repeated instances have come before my notice, where grave-diggers have been attacked on the spot with vomiting and subsequent diarrhœa, owing to the foul effluvia, particularly in cases where fœtid water had escaped suddenly from a neighbouring grave or vault. This accident sometimes greatly adds to the evils of surcharged burial grounds, as the putrid fluid is thrown out on the surface, and, as I have known, to the extent of forty buckets from one grave. The following is an instructive case, and exactly corresponding to many that have been recorded by authors. Three workmen were engaged in repairing a church, which I had occasion to inspect; they had placed their ladder in the aisle, the floor of which gave way, and crushed in a coffin in a vault beneath. There was a large escape of offensive effluvia, which caused immediate sickness and vomiting. This was followed by fever in all the men, one of whom died.

My own observations have satisfied me that, in addition to the dangers suddenly arising, as in the instance just adduced, the men who are constantly employed in large cemeteries, in contradistinction to grave-diggers only occasionally occupied, suffer, like the slaughtermen noticed by Professor Owen, in their general health, from the nature of their employment: they are pallid, cadaverous, and of a sickly aspect. The celebrated Fourcroy makes the same remark, adding that these men present all the symptoms which announce the action of a slow poison.

Occurrences of the kind I have adduced are decisive of the deleterious action of graveyard effluvia, in the case of persons exposed to them in a concentrated form; and no objections of a negative character, often most vague in themselves, can suffice to disprove them. That the inhabitants of houses closely surrounding overcrowded churchyards may also suffer in their health, I have no doubt. The actual condition of such burial places is in itself sufficient proof. On examination, it is found that, for three or four feet, the soil, whatever may have been its original character, consists of dark, loose mould, composed of earth and organic matter, and continually receiving the poisonous gases and effluvia generated beneath. Where a large number of corpses have been piled up in deep graves, according to the old vicious system, the most offensive smell is perceived by passers by, especially in hot and muggy weather; of this fact I have frequently received medical evidence. If the black mould I have referred to be collected in a closed room, it emits a foul stench; and, if spread about near dwellings, it may cause, like night-soil under similar circumstances, a sudden outbreak of disease. Of this a striking instance was a few years since afforded in a country town, where the ancient churchyard having been raised, as so often happens, owing to repeated burials, it was lowered by the removal of some hundred loads of the rotten earth; this being partly applied to the gardens as manure, and partly stored up for use near a number of cottages. As always happens under like circumstances, the soil became more offensive from exposure and disturbance; when suddenly, after a period of warm wet weather, the wife of the clergyman, in whose garden the soil had been used as a top dressing, was seized with low fever and died, and one or two of the children were similarly attacked. The fever next broke out in the cottages where the soil had been applied to the gardens; and, last of all, in the dwellings near the large heap, after the wind had for some time blown from that direction. The gentleman who attended the sufferers, and who was an intelligent practitioner, had the firmest conviction that the epidemic was owing to the emanation from the churchyard matter.

Time will not permit me to notice other sources of effluvia, or I should particularly have desired to call attention to the great evils inflicted on the public health by certain offensive trades, such as bone boiling, gut spinning, artificial manure manufactories, etc.; in all of which fœtid effluvia are given off so abundantly as to poison the air of the whole neighbourhood. On this point I have received a large amount of medical evidence, all corroborative of the sickness thus directly caused; in fact, I never conversed with a medical man, practically acquainted with such localities, who was not convinced of the direct noxious influence of these emanations. At the present period, when legislative efforts are being made to rid populous neighbourhoods of these serious evils, such testimony is of great value; nor should the proceedings of other countries, in regard to the regulation of offensive trades, be without their weight, when it is recollected that, as in France, for example, they were based on the careful and deliberate investigations of some of the most distinguished physicians and chemists of Europe.

Before concluding this address, I beg to submit a few considerations respecting the *modus operandi* of the deleterious agents of which we have been speaking. Being in a subtle gaseous form, there can be no dispute that they gain direct access to the blood; nor that, whatever may be the mode in which their ultimate effects are manifested, they primarily affect the condition of that fluid. This being so, some time must elapse before the mischief displays itself, though this may be brief, as where vomiting is excited by exposure to a putrid corpse. It has indeed happened, that persons in descending into tombs and pits, where many bodies were deposited, have on the instant become insensible, occasionally convulsed, and then perished, of which an instructive example is given by Haquenot, in the *Dictionnaire Philosophique;* but here, as it would appear, the mischief depended on asphyxia, caused by the accumulation of carbonic acid. Late researches have apparently established as a principle, that all decomposing protein compounds act as ferments on the blood; and this may be assumed, also, in regard to animal putrescent effluvia. The interesting observations of Dr. Carpenter further tend to show, that the well-known constant presence of decomposing organic substances in the circulating fluid, promotes the action of similar matter introduced from without. These views appear to me so well established, that, in consideration of the great importance of the subject, I would trespass for a moment on your patience, whilst I allude to the large amount of metamorphosed matter which must exist in the blood, resulting from the enormous activity of the vital processes, and which is not yet thoroughly comprehended. Of late several attempts have been made to determine this more precisely than before; for instance, it has been estimated that there passes by the thoracic duct in twenty-four hours an amount of matter equal to the whole quantity of the blood; that from ten to twenty-two pints, according to the observer, of gastric juice are secreted per diem. These are given simply as indications, and are probably greatly exaggerated, especially the latter. But it is certain the act of secretion requires a large quantity of liquid as a condition; how much water, for instance, is required to carry out of the blood the small amount of solid matter of the urine, and this independent of mere excess of liquid ingesta. The mutation of solid parts is also most active, as of the epithelia, evidenced equally by their *débris,* wherever they are shed, and by the reproductive process on the deep parts of the mucous surfaces, epidermis, and glands. The valuable researches of Professor Graham on osmotic force have, for the first time, satisfactorily explained all this destruction, by proving that no current can take place across an organic membrane—a cell, for example—without this last becoming decomposed; thus illustrating one of the most profound questions of the animal economy, by demonstrating the conversion of one great force in nature, the chemical, into another, a mechanical power, or motive force. The same principle is in operation in apparently the most permanent solids, though, as the change is molecular, and the form remains, it usually escapes detection. As illustrations, I would refer to the metamorphoses of a developing bone, which is changed again and again before it is formed; and to the casting off of the mucous coat, and the probable disintegration and absorption of even the whole of the developed muscular fibres of the uterus, after each gestation.

As chemical action and consequent organic disintegration are thus the conditions of all vital action, it is easy to perceive that in childhood, when every organ in the body is undergoing rapid and repeated metamorphoses, in its progress towards completion, an enormous amount of altered matter enters the blood; and when to this is superadded the large quantity of ingesta, gaseous, liquid, and solid, some conception of the actual condition of the circulating fluids in youth may be formed. Under favourable conditions, and a large supply of the great purifier, oxygen, all goes on well; but, for the reasons stated, young growing children, and above all infants, must be regarded as being in a most susceptible state, ever liable to be injuriously acted on by the introduction of noxious gases and putrid emanations from without. That this is the true explanation of the most potential cause inducing the enormous infantile mortality of crowded and unhealthy localities, will scarcely be doubted by those who have carefully studied the question.

But to return to the mode of action of animal effluvia. One of their most constant results, as we have seen, is gastric and intestinal disturbance, inducing what is often considered as ordinary dyspepsia, vomiting, and diarrhœa. The latter doubtless depends on the poisoning of the blood, inducing, as a specific result, an increase of that eliminating process, which is a normal action of the intestinal mucous membrane; an explanation which, among other considerations, is borne out by the fact observed by Dr. Brinton and others, that the peculiar odour characterising effluvia, may sometimes be detected in the stools. His view of the subject is the more important, since, as it seems to me, it brings the agent in question into the same great category as those destructive epidemics which are characterised by fluxes from the bowels.

That during epidemic seasons these effluvia predispose to fever of a gastric type, is rendered probable by the history of many such outbreaks that have been investigated by myself and others in late years.

It also cannot be doubted, that in the crowded abodes of the poor in large towns, the animal matter excreted from the lungs and skin give off emanations, which predispose to low fever and cholera; as indicated by the facts, that of all the deleterious conditions prevalent in such localities, overcrowding is the most constantly present; and that the most destructive outbreaks of cholera have been traced directly to that as a cause.

In circumstances where persons are exposed to the continued operation of animal effluvia, the general powers of the system invariably suffer, of which the condition already noticed of slaughtermen and the grave-diggers of large cemeteries may be received as a specimen.

In the remarkable case of Christ Church workhouse, Spitalfields, recorded in my report on cholera, the nutritive actions were so much disturbed by the respiration of putrid effluvia, that in three months twelve infants became victims of gangrenous ulcers of the mouth and genitals.

In conclusion, I would remark, that in some of the effects induced, the agents we have been considering seem to be the immediate cause of the morbid condition, such as diarrhœa and vomiting, and the depressed state of the system. When concerned, however, in the production of fever, it may be inferred that they only operate as predisposing causes, though the inquiry relative to the primary or essential cause of epidemic disease, the *materies morbi*, is still most obscure. All theories regard it as an entity distinct from the predisposing causes; and yet this doctrine, as indeed do all others in the present state of our knowledge, leaves much that is inexplicable. In the case of what may be called the great epidemics, as plague, the black death, cholera, it has been usual to consider the essential cause as some kind of morbific influence, that travels from east to west, whether this be what is vaguely called atmospheric, electric, or telluric; a certain something that is locomotive, in contradistinction to endemic. And the accurate history of all great epidemics has on the surface everything to corroborate this idea: we see these great pestilences slowly progressing from country to country; marching in a certain course; often recurring after an interval of years, greater or less, attacking as in a normal order certain countries, towns, and districts. In the great outbreaks, which arrest by their frightful mortality the attention of whole nations, and engage the pen of the historian, and thus in their details have been transmitted to us, all seems to fall in with the locomotive theory. But when we ourselves have an opportunity of investigating the matter—and happily the only two members of the universal epidemics now presented to our scrutiny in this country are cholera and influenza—it is found that, besides the great periodic outbreaks following a definite track, there are invariably occurring, from time to time, isolated or sporadic cases; and these exceptional cases, as they are termed, as in all like investigations relative to natural phenomena, are for the elucidation of the general law so many delicate tests, like malformations in the general law of development. How do these occur, and how are they caused? That they do occur is certain, Since the first appearance of cholera in 1832, and especially since the great epidemic of 1848-9, sporadic cases of true malignant cholera have been observed by those well acquainted with the disease in various parts of England, a multitude of which instances have been related to me. Now, how are these scattered cases, separated from each other by large distances and intervals of months or years, to be explained? Can it be imagined that, thus restricted, they depend on some general progressive atmospheric or terrestrial influence? The circumstances seem to forbid such an hypothesis.

These, and many other moot points, remain for future investigation. In the mean time, the so-called predisposing causes of zymotic disease are open to our scrutiny; and as these are, moreover, capable of great reduction, and in many instances of eradication, it is to them that the steady attention of all engaged in sanitary inquiries should be directed.

